# Myths and beliefs about insulin therapy in patients with diabetes mellitus and their family caregivers from a hospital in northern Peru, 2020

**DOI:** 10.17843/rpmesp.2023.401.12210

**Published:** 2023-03-30

**Authors:** Jhoana Lorena Vera Ubillús, Blanca Katiuska Loayza Enríquez, Rosa Elizabeth Guarníz Lozano, Franco Ernesto León Jiménez

**Affiliations:** 1 Quirichima Healthcare Center, Lambayeque, Peru. Quirichima Healthcare Center Lambayeque Peru; 2 San Martín de Porres University, Filial Chiclayo, Chiclayo, Peru. San Martín de Porres University San Martín de Porres Universitys Filial Chiclayo Chiclayo Peru; 3 Católica Santo Toribio de Mogrovejo University, Lambayeque, Peru. Católica Santo Toribio de Mogrovejo University Católica Santo Toribio de Mogrovejo University Lambayeque Peru; 4 Vice-Rector's Office for Research Norbert Wiener Private University, Lima, Peru. Norbert Wiener Private University Vice-Rector's Office for Research Norbert Wiener Private University Lima Peru

**Keywords:** Insulin, Diabetes Mellitus, Qualitative Research, Belief, Group Interviews

## Abstract

**Objective.:**

To analyze and explore the myths and beliefs about insulin therapy in patients with diabetes mellitus and their family caregivers from a general hospital in northern Peru in 2020.

**Materials and methods.:**

This qualitative study used a thematic analysis model, following the interpretative paradigm. Sociodemographic and clinical data were obtained from medical records. Patients with diabetes that used some type of insulin for at least three months prior to the study were interviewed, as well as their family caregivers. Patients participated in a focus group and in-depth interviews; family caregivers participated only in in-depth interviews.

**Results.:**

Twelve patients with diabetes (11 with type 2 diabetes mellitus) were included; six in the focus group and six in the in-depth interviews. Seven family caregivers were included. After analysis, we obtained four categories: 1) beliefs related to starting insulin treatment: treatment of choice after failure of other drugs, cures diabetes, regulates sugar, fear of injectables; 2) beliefs related to treatment adherence: decompensation for not using insulin, insulin is necessary to live; 3) beliefs related to alternative therapies and cost: use of alternative therapies, high cost of insulin; and 4) myths related to the use of insulin: generates dependence, dependence for insulin administration, negative effects of insulin.

**Conclusions.:**

The beliefs and myths of patients treated with insulin arise from the beginning of treatment, remain throughout the course of treatment, and are often reinforced by the worldview of family members.

## INTRODUCTION

Diabetes *mellitus* (DM) is a public health problem. According to the estimates by the International Diabetes Federation (IDF), approximately 537 million people were living with DM in 2021 worldwide, with a projection of 643 million by 2030 and with direct costs close to one trillion dollars. The prevalence of DM in Peru is 5.9% (95% confidence interval: 5.5-8.2), and approximately 90% of patients have type 2 DM (T2DM) ^1^.

Insulin is an important component of the treatment in patients with T2DM. Approximately 13-26% of these patients are estimated to use some type of insulin as part of their treatment ^2^^,^^3^. The American Diabetes Association (ADA) recommends the use of insulin in patients with uncontrolled disease (weight loss, glycosylated hemoglobin greater than 10% or random glycemia greater than 300mg/dL) ^4^.

Adherence to treatment is essential in patients requiring insulin, however, it is not optimal. In 2013, a systematic review by Davies *et al*. reported that the frequency of adherence ranged from 43 to 86% ^5^. Flores conducted a descriptive study in Lambayeque, Peru with 100 people diagnosed with diabetes during 2020 using the Morinski Green test, and found that the frequency of non-adherence was 29% ^(^^6^. This situation leads to poor glycemic control and clinical complications with increased mortality ^7^.

Current literature supports the fact that structured educational programs can have an impact on people with T2DM who use insulin, improving metabolic control, without producing hypoglycemia. Hermmans *et al*. carried out an open-label clinical trial during 2017 to assess the effectiveness of a self-management-oriented educational program compared to standard education and found improved glycemic control without increased hypoglycemia ^8^. Likewise, Yorke *et al*. conducted a systematic review of 36 studies including 11,880 patients in 2017, and reported that educational and structured interventions reduced morbidity and mortality and hypoglycemia events ^9^. On the other hand, in 2017, a systematic review by Iquize *et al*. found that structured education improved quality of life, but not glycosylated hemoglobin values ^10^. This discrepancy could be due, among other reasons, to different perspectives and opinions on insulin use in patients ^11^.

The perspective of the patient and family caregivers must be taken into account in order to ensure the success of educational interventions, particularly in patients with multiple comorbidities and target organ damage. Understanding what patients and caregivers think, feel and what myths and/or beliefs they have regarding their disease and particularly those related to the medication is crucial. Educational programs, for the most part, do not consider this aspect. Quali-quantitative studies have shown that, by identifying myths and beliefs early, educational strategies aimed at improving glycosylated hemoglobin levels can be developed ^12^^-^^14^. These aspects along with the subcutaneous application, dosage calculation and adverse events related to insulin may cause the patient and family not to accept treatment ^15^. It should be considered that fears, expectations, myths and beliefs may vary from one context to another. On the other hand, family support in a chronic and disabling disease such as T2DM is fundamental. However, caregivers face a complex reality: difficulties in understanding the disease, beliefs, educational level, feelings of uncertainty and the health system’s failure to provide support for healthy lifestyles ^16^^,^^17^.

Due to the lack of data on the perspective of the patient and family caregiver, it is important to obtain useful information to improve the chronic noncommunicable disease program in public hospitals in Peru. Therefore, this study aimed to analyze and explore the myths and beliefs about insulin therapy in patients with T2DM and their family caregivers in a hospital in northern Peru in the year 2020.

**KEY MESSAGES**

Motivation for the study. Adherence to insulin treatment in diabetic patients is suboptimal. Determining the clinical and sociodemographic factors is necessary to understand the patient’s perspective on treatment.

Main findings. Participants expressed the following ideas about insulin treatment: it is necessary to live if oral medications fail, it cures diabetes, it regulates sugar. In addition, fear of needles, becoming dependent, and hypoglycemia were also reported. The high cost of insulin limits its use and medicinal plants are perceived as an alternative.

Implications. The perspective of the patient and family members are fundamental for diabetes education.

## MATERIALS AND METHODS

We used the Synthesis of Recommendations by the 2014 Academy of Medicine Standards for Reporting Qualitative Research to report our methods and results ^18^.

### Design

This research was a thematic analysis conducted with a qualitative approach, interpretative paradigm, and was carried out in three stages: description, reduction and interpretation. The focus groups moderator and the person responsible for the in-depth interviews (BLG) was a graduate in nursing, qualitative researcher, university professor, with no relationship with the patients or family caregivers. The person in charge for taking notes and recording the interviews was a human medicine intern (LVU), who had no relationship with the patients and was trained by the moderator.

We defined myths and believes according to Taipe and Diez, respectively. Taipe defined myth as a social construct shared by different individuals that is spread by society; it lacks a specific author and tries to explain a phenomenon without scientific support, but since it becomes part of the culture, members of the society tend to consider the myths as being true. Taipe also considers myths to be the opposite of truth and reality and that they try to explain something outside rationality ^19^. According to Diez, beliefs are reflexive ideas assumed by society, which individuals consider to be true and are adopted as an interpretation of reality; there are objective reasons to consider them as such ^20^.

### Setting and study population

The study was conducted in the endocrinology service of the Hospital Belén de Lambayeque, of the Ministry of Health (MINSA) of Peru, which is a medium complexity healthcare facility in the province and department of Lambayeque in northern Peru, which treats patients with diabetes. The study was carried out between January and March 2020.

We included patients with type 1 diabetes *mellitus* (T1DM) and T2DM who were selected by convenience through thematic saturation. Patients using any type of insulin (rapid-acting, intermediate-acting and long-acting) for at least three months prior to the study were included. On the other hand, patients with proven cognitive impairment, acutely ill and with other major chronic diseases (tuberculosis, human immunodeficiency virus) were excluded. Family members who lived with the patients and were responsible for taking them to their check-ups were included.

### Data collection techniques and instruments

Data regarding sociodemographic characteristics, glycosylated hemoglobin and fasting glycemia were collected from medical records (Supplementary Material). Two open-ended question guides were developed and reviewed by an internist and a qualitative researcher. The first interview/focus group guide of six open-ended questions served as a guide for the patient focus group and the in-depth interviews with the other group of patients (Supplementary Material). The second interview guide consisted of three open-ended questions and was used during the in-depth interviews with family members (Supplementary Material).

### Procedures

Participants were selected after their outpatient appointment in the endocrinology department. The focus group was carried out first, in which patients with hearing loss, blindness or mobility difficulties could communicate. The interview took place in a closed office and with the support of the patient’s relatives, who helped them answering the questions. The session lasted 60 min and was recorded using a smartphone voice recorder. In addition, the recording as well as field notes were later included in a single file (LVU). On the other hand, the in-depth interviews with patients lasted 60 min each. The data collection and transcription of the in-depth interviews were carried out in a similar way to the focus group.

### Data analysis

A code was assigned to each patient and family interview as follows. Patient: Capital P followed by an Arabic numeral. Family member: F followed by an Arabic numeral. Initially, the audio recording process was carried out by one of the authors (BLG), with expertise in this process.

Data was processed manually, following the phases of the thematic analysis method proposed by Braun and Clarke, which includes identifying, analyzing and reporting patterns (themes) within empirically collected data. It minimally organizes and describes in detail the data set and interprets aspects of the research topic. In other words, it involves searching through a data set to find repeated patterns of meaning ^21^. The phases were: transcription, text reduction and discovery phase, which resulted in defining study “themes”. Then, data was classified according to their common content and linkage to one of the themes. Finally, during the coding phase relevant fragments were associated to a category of a theme by means of a code (number). Coding and categories were redefined and adjusted to the data during the analysis. Data from patients’ and caregivers’ discourse were triangulated. This work was carried out by one of the authors (BLG).

### Ethical Aspects

The project was approved by the Teaching Department of the Hospital Provincial Docente Belén de Lambayeque and the Research Ethics Committee of the Faculty of Medicine of the Universidad Católica Santo Toribio de Mogrovejo (Resolution No. 573-2019-USAT-FMED dated October 31, 2019), with an amendment to the document by Resolution No. 017-2022-USAT-FMED. The informed consent form provided to patients and family caregivers were different but both included the objective of the study, as well as the information confidentiality and the free participation statements. Finally, participants received information on myths and beliefs about diabetes and insulin therapy. A copy of the final report was given to the hospital’s training office.

## RESULTS

Twelve patients with diabetes (11 with T2DM) were included; six patients participated in the focus group and six patients in the in-depth interviews. Seven family caregivers were also interviewed. In addition to quantitative information, we found that, of the total 12 patients, six had retinopathy, two had nephropathy, one had a previous amputation and two had peripheral neuropathy. No patient received dialysis. Quantitative data are shown in Table 1: age, time of illness, years receiving insulin, fasting glycemia and glycosylated hemoglobin.

**Table 1:** Characteristics of included participants.

Characteristics	Total (n=12)	%
Women	10	84.0
Type 2 diabetes *mellitus*	11	91.6
Educational level		
Primary school	3	25.0
Secondary school	9	75.0
Macrovascular complications	3	25.0
Microvascular complications	7	58.3
Type of therapy		
Baseline	6	50.0
Baseline plus	3	25.0
Baseline bolo	3	25.0
Insulin type		
NPH	6	50.0
NPH and crystalline	6	50.0
Responsible for applying insulin		
Patient	6	50.0
Family member	6	50.0
Age ^a^	51.5	42.5-58.0
Years with diabetes ^a^	7	5-13
Fasting blood glucose (mg/dL) ^a^	170	150-186
HbA1c (%) ^a^	9	8.2-10.0
Months with insulin therapy ^a^	24	5-72

a median and interquartile range.

NPH: intermediate-acting insulin; crystalline: rapid-acting insulin; HbA1c: glycosylated hemoglobin.

Likewise, four categories were systematized according to the time of insulin therapy: 1) beliefs related to the initiation of insulin treatment; 2) beliefs related to treatment adherence; 3) beliefs related to alternative therapies and cost; and 4) myths about the use of insulin (Table 2).

**Table 2:** Categories identified in the text analysis.

Category 1. Beliefs related to treatment initiation.
*It is the treatment of choice after other drugs failed.*
*It cures diabetes*
*Regulates blood sugar*
*Fear of needles*
Category 2. Beliefs related to treatment maintenance
*Fear of decompensation for not using insulin*
*Insulin is necessary to live*
Category 3. Beliefs related to alternative therapies and cost
*Alternative therapies provide same benefit as insulin*
*Insulin is expensive*
Category 4. Myths related to the use of insulin
*Dependence on insulin*
*Creates dependence*
*Negative effects*

### 1. Beliefs related to the initiation of insulin treatment

The interviewees considered insulin to be a second step in the treatment of their condition, usually preceded by failure with oral antidiabetics. The high cost of the insulin analog and the need to use insulin were also mentioned:

*I started with insulin because the pills did not lower my glucose. I started with insulin <trade name> and because of the cost I switched to NPH. In patients with type 2 diabetes insulin is used when the pills no longer work. My glucose was too high despite taking pills and because I was messing with food and drinking alcohol* (P4).

Diabetes is a chronic disease for which there is still no definitive treatment. Treatment alternatives slow progression and reduce target organ complications. However, one patient in the focus group mentioned that insulin could cure the disease:

*My daughter says that insulin is a medicine that replaces the pancreas and kills the disease* (P6).

This belief was also mentioned by a family member:

*Insulin is a vitamin so that can cure you from diabetes* (F7).

Family members play a fundamental role in the support or rejection of the drug, and their opinions or judgments can influence the start of treatment:

*My husband listened to me regarding the fact that insulin is for his own good because we have been married for 45 years, I told him that if he is prescribed insulin it is for his own good and he should be calm* (F1).

Patients and family members mentioned that insulin can control glycemia levels:

*I have used insulin for 10 years. It is a drug that regulates sugar, it is necessary for the liver to process it from blood and food* (P7).

*Insulin helps lower glucose when it is too high* (F6).

Another important finding was the fear of injectables, which could condition the start of treatment and the need for a family caregiver to help them when applying the medication:

*At the beginning I was afraid because the needles with which they put serum in you are big. But insulin needles don’t hurt because they are small. I would like insulin not to be injectable but in syrup because it is easier, I don't have to keep it in ice or soak it* (P6).

We also found perceived difficulties in getting an injection at the beginning of treatment:

*At the beginning I did not know how to inject myself, they did not want to put it even at the pharmacy. Later I had to learn, my niece taught me, because I did not want to bother others* (P8).

Family members also showed fears at the beginning of treatment:

*I was afraid at the beginning because I see many disabled patients, and if they are not cured, they cut off their legs* (F3).

The patient’s correct or erroneous beliefs remained during treatment; therefore, we created a category that encompasses the different beliefs with which they coexist and with which they manage their treatment.

### 2. Beliefs related to treatment adherence

Adhering to insulin therapy is a challenge for people with diabetes. Some patients were afraid of decompensation if they discontinue treatment:

*I am afraid that they will not inject me and I will get sick* (P1).

Patients trust the treatment, accept it and believe that it helps them to live better:

*The doctor told me that I could stay on insulin for the rest of my life and I accepted it calmly because as long as I can stay alive there are no problems* (P5).

### 3. Beliefs related to alternative therapies and cost

Regarding alternative therapies, some interviewees reported benefits and the belief that it can replace insulin:

*I usually take care of myself with pills and natural products like Spirulina and Ganoderma mushroom coffee. These products are equal to insulin because they control me and lower my sugar. I don't think this is my case because this natural product is enough. At some point I will stop the insulin and just take the natural* [*product* ] *because they do the same action* (P3).

*There are more natural remedies that are the same, plants that they sell in the market. That way I wouldn’t have to inject myself all the time* (F6).

However, others mentioned having tried them and stopping shortly after because they did not perceive any improvement:

*I have used yacon to lower glucose, but even taking that and other natural remedies did not work for me* (P10).

Some patients mentioned that they were prescribed insulin, but the hospital did not have it in stock, so they had to buy it and the cost was very high:

*I would like to stop using insulin because it costs a lot* (P3).

Family members share the same argument:

*Insulin is expensive, I buy it because sometimes there is none in the SIS* (Comprehensive Health Insurance) (F7).

### 4. Myths related to the use of insulin

This section shows the discourses of patients and family members that fall into the category of myths:

*I prefer pills because they are easier. Sometimes I am afraid to give him insulin, or that his blood will leak or he will get sick* (F4).

Patients were of the opinion that they could develop dependence, because when they stop using insulin, they experienced discomfort.

*Insulin can cause dependence, because when you stop using it you feel that you have to take it again to be well* (P9).

Participants expressed their desire to stop treatment as the disease progressed, because the more years with diabetes, the less effect the medication has:

*Insulin acts up to a certain point until the body deteriorates as a result of the disease* (P11).

Patients confuse symptoms of disease progression due to target organ damage with adverse events of the medication:

T*hey say that insulin is harmful and damages organs such as the eyes* (P8).

Information provided by the physician can help to improve adherence:

*At the beginning I was afraid that they would damage something like my stomach, but then the endocrinologist told us that it would not hurt me because the needle is going to reach the fat precisely* (P4).

Hypoglycemia is a frequent acute complication related to inadequate insulin doses, associated oral antidiabetics, deficient caloric intake, progression of renal failure or infectious processes. Many times, the fear of this condition causes the patient to administer lower doses than usual:

*I have had hypoglycemia as a problem with insulin. That is why I stopped using it for a while, but then I was tested and started using it again. I think I get low blood sugar when I exercise and don’t eat well* (P12).

## DISCUSSION

The interviewed patients, despite being people who have diabetes with complications, were not aware of the fundamental aspects of their own care and control, such as the use of insulin. Therefore, we found several myths and beliefs regarding its use.

The idea that the disease can be cured was found in 11.7% of people with diabetes receiving first-level care, as reported in a study from Mexico in 2018 ^22^, and is a similar figure to that found by León-Jiménez in a study conducted in patients from northern Peru in 2020 ^23^. However, this belief was not found by a mixed study in people with diabetes from the Lambayeque Social Security in 2017 ^24^. We did find this belief in our studied population, despite having been explored in similar scenarios. These findings depend on multiple factors such as educational level, hospital level, time of illness and other characteristics.

Likewise, we found that some patients considered discontinuing treatment because they believed insulin was related to blindness, renal failure and even limb amputation; they believed these conditions were not part of the disease progression. This finding is similar to the results of other studies such as a systematic review by Ng *et al*. ^15^. This finding shows the limitations of public hospital educational programs in making patients aware of their disease. The ideal education is structured, sequential, scheduled and measurable, in groups or individually ^12^. It is necessary for patients to understand that treatment improves their lives and delays complications, as it emphasizes the importance of diabetes education. In Peru, this aspect is still underdeveloped and there is no standardized and systematic form of application.

The fact that patients know that insulin regulates blood sugar levels is positive and encouraging. This result is similar to what was reported by Tan *et al*. in patients who did not respond to oral therapy in the Asian region in 2003, 59.7% participants mentioned that insulin controlled their glycemia ^25^. Likewise, a qualitative study by Jenkins *et al*. in an English population in 2010, reported that patients accepted the use of insulin more easily as their disease progressed ^26^. This concept was also evidenced in our study. The so-called psychological resistance to insulin is a concept found in those who have failed to control the disease and consider it as their last hope ^27^. Brod *et al*. in a mixed study conducted in five countries (Germany, Sweden, the Netherlands, the United Kingdom, and the United States) found that patients considered insulin as a last-resort medication and they consider using it as a personal failure ^14^. This conception is not adequate, because although it is true that insulin is indicated in decompensated patients, other patients benefit from its use from the beginning^4^.

It is possible that education regarding diabetes, which is still lackluster in Peru, does not guide patients in the early use of insulin. There are studies that show that not only patients, but also health personnel, including physicians, face barriers when using insulin. This fact was evidenced in the systematic review by Ng *et al*. in 2015, who evaluated poor knowledge and skills (nine studies), physician inertia (five studies) and language barriers (four studies) ^15^. However, this possibility was not explored in our study.

The participants described feeling fear of decompensation due to not using insulin. We did not find any other studies reporting this belief. Although it is not a barrier, it should be addressed by health personnel. Patients who consider insulin to be a useful tool adhere more frequently to treatment ^25^.

The concept of becoming insulin dependent has also been reported by other studies. Nakar *et al*. in a case-control study involving 103 patients, found that the group in need of insulin compared to those already receiving insulin had a greater fear of insulin addiction (39% vs. 20.8%; p<0.01) ^28^. This is a belief that should be addressed early on.

Participants reported losing autonomy and depending on others, which has been found by other studies as well ^16^^,^^17^. Patients should be taught that having the assistance of a family member is not a limitation but an advantage for them. The mental and emotional evaluation of patients and their caregivers is fundamental ^17^^,^^23^. Family support is essential in cases of blindness and functional limitation due to neurovascular sequelae.

Most participants initially rejected insulin because it is injectable. Fear of needles has been identified as a barrier to insulin use in several studies ^16^^,^^29^. There is evidence that this concern tends to disappear over time ^14^.

Fear of hypoglycemia was described by our patients. It is a frequent event; therefore, patients and their family caregivers should be informed about it. This fear is valid and has been reported by multiple studies ^29^^-^^31^. At discharge from hospitalization, health care personnel should ensure that the patient/family member learns the exact dosage, forms of insulin application, and alarm symptoms of hypoglycemia. LaManna *et al*. in a systematic review of eight studies published between 2014 and 2017 found that personalized education had an impact on the number of hypoglycemia events and their symptoms ^31^. We do not know if there is a structured protocol for education in this hospital or in others.

On the other hand, patients’ uncertainty leads them to use alternative therapies. Ramirez *et al*. conducted a descriptive study in Mexico in 2021, in which they reported that 21% of patients used traditional medicine, the most frequent being herbal tea ^32^. The mention of *ganoderma* in our study is striking. *Ganoderma lucidum* has been shown to have proteoglycans and other components with a hypoglycemic effect ^33^; the same is true for spirulina and diabetes. In fact, there is a systematic review of nine studies that mention its beneficial effect on lipids and fasting glycemia ^34^. Experimental studies in animal models have shown evidence of the antioxidant and hypoglycemic effect of yacon (*Smallanthus sonchifolius*) ^35^.

In addition, we found concerns regarding the costs of insulin and its use. The Peruvian Comprehensive Health Insurance includes NPH insulin (Neutral Protamine Hagedorn) and crystalline insulin in its guidelines. However, participants mentioned that those types of insulin were not always available and had to be purchased. Other participants used insulin types that were not included in the national program as well as pre-filled insulin devices. While it is true that hypoglycemia is slightly less frequent with these insulin types, there is no difference in the efficacy of controlling diabetes, in the occurrence of micro- or macrovascular complications, or in mortality ^36^. Additionally, the ADA does not consider them as the first-line medication ^4^.

We also found concerns about glycemia monitoring. An alternative is to strengthen the use of telemedicine as a powerful monitoring tool in primary care, which has proven to be effective^37^. Many patients or caregivers now have smartphones.

The methodological limitations of the study include the small number of patients with T1DM and the large number of women. Although 90% of patients with DM are type 2 ^1^, it is necessary to carry out a study on patients with T1DM, which is more frequent in young people and children. This group of patients, as well as their parents, mostly use insulin, so it would be important to explore their myths and believes as well. On the other hand, more men should be included in future studies because their way of thinking about their disease is different from that of women ^38^. Other limitations include: having only one focus group instead of two or more, in order to consider a better spectrum of participants (sex/time of illness), the questions applied to patients in the in-depth interviews were the same as those of the focus group, data was interpreted by only one researcher and the credibility criterion (in which the results are contrasted with the participants) was not met. Finally, we should mention that our results were obtained before the COVID-19 pandemic. The patient/family worldview might have changed with this new context, which should be explored. In addition, several MINSA healthcare strategies was affected by the pandemic, especially those regarding chronic diseases ^39^.

In conclusion, the myths and beliefs regarding insulin therapy appear from the beginning of treatment and are maintained during treatment, often being reinforced by the worldview of family members. These myths and beliefs reinforce the importance of structured and proactive education, by understanding that behind each patient there are different concepts about the health and disease process, and that they may be different from those of the healthcare personnel. Not understanding the myths and beliefs can have an impact on the adherence to treatment, their relatives and on the cardiovascular prognosis of patients.

## References

[1] International Diabetes Federation (2021). IDF Diabetes Atlas 2021.

[2] Jiménez-Corona A, Aguilar-Salinas CA, Rojas-Martínez R, Hernández-Ávila M (2013). Diabetes mellitus tipo 2 y frecuencia de acciones para su prevención y control. Salud pública Méx.

[3] Pinto Valdivia M, Guevara Linares X, Huaylinos Párraga Y, Chia Gonzales S, Manrique Hurtado H (2019). Características clínicas y epidemiológicas de los pacientes adultos con diabetes tipo 2 tratados con insulina en un hospital general de Lima. Rev Soc Peru Med Interna.

[4] ElSayed NA, Aleppo G, Aroda VR, Bannuru RR, Brown FM, Bruemmer D (2023). 9 Pharmacologic Approaches to Glycemic Treatment: Standards of Care in Diabetes-2023. Diabetes Care.

[5] Davies MJ, Gagliardino JJ, Gray LJ, Khunti K, Mohan V, Hughes R (2013). Real-world factors affecting adherence to insulin therapy in patients with Type 1 or Type 2 diabetes mellitus a systematic review. Diabet Med.

[6] Flores A (2022). Factores asociados a la no adherencia a insulinoterapia en pacientes con diabetes mellitus 2 de un hospital de Lambayeque 2019-2020.

[7] Fu AZ, Qiu Y, Radican L (2009). Impact of fear of insulin or fear of injection on treatment outcomes of patients with diabetes. Curr Med Res Opin.

[8] Hermanns N, Ehrmann D, Schall S, Maier B, Haak T, Kulzer B (2017). The effect of an education programme (MEDIAS 2 BSC) of non-intensive insulin treatment regimens for people with Type 2 diabetes a randomized, multi-centre trial. Diabet Med.

[9] Yorke E, Atiase Y (2018). Impact of structured education on glucose control and hypoglycaemia in Type-2 diabetes a systematic review of randomized controlled trials. Ghana Med J.

[10] Iquize RCC, Theodoro FCET, Carvalho KA, Oliveira M de A, Barros J de F, Silva AR da (2017). Educational practices in diabetic patient and perspective of health professional: a systematic review. J Bras Nefrol.

[11] Brod M, Kongsø JH, Lessard S, Christensen TL (2009). Psychological insulin resistance patient beliefs and implications for diabetes management. Qual Life Res.

[12] Millian A (2015). Myths and Misconceptions about Insulin Therapy among Latinos/ Hispanics with Diabetes A Fresh Look at an Old Problem. J Diabetes Metab.

[13] Baghikar S, Benitez A, Fernandez Piñeros P, Gao Y, Baig AA (2019). Factors Impacting Adherence to Diabetes Medication Among Urban, Low Income Mexican-Americans with Diabetes. J Immigr Minor Heal.

[14] Brod M, Alolga SL, Meneghini L (2014). Barriers to Initiating Insulin in Type 2 Diabetes Patients Development of a New Patient Education Tool to Address Myths, Misconceptions and Clinical Realities. Patient.

[15] Ng CJ, Lai PSM, Lee YK, Azmi SA, Teo CH (2015). Barriers and facilitators to starting insulin in patients with type 2 diabetes A systematic review. Int J Clin Pract.

[16] Pinzón-Rocha ML, Aponte-Garzón LH, Hernández-Páez RF (2012). Experiencia de los cuidadores informales en el manejo de la diabetes mellitus tipo II. Orinoquia.

[17] Amparo D, Rico P (2013). Características sociodemográficas asociadas a la sobrecarga de los cuidadores de pacientes diabéticos en Cúcuta. Rev Cuid.

[18] O'Brien BC, Harris IB, Beckman TJ, Reed DA, Cook DA (2014). Standards for reporting qualitative research a synthesis of recommendations. Acad Med.

[19] Taipe N (2000). Los mitos Consensos, aproximaciones y distanciamientos teóricos. Gaz Antropol.

[20] Diez Patricio A (2017). Más sobre la interpretación de ideas y creencias. Rev Asoc Esp Neuropsiq.

[21] Braun V, Clarke V (2006). Uso del análisis temático en psicología. Investigación Cualitativa en Psicología.

[22] Salazar Fonseca E, Ponce Rosas E, Jiménez Galván I, Azucena Cervantes NA, Jiménez Hernández J (2018). Mitos y creencias sobre la diabetes en pacientes de una unidad médica de atención primaria en la Ciudad de México. Archivos en medicina familiar.

[23] León-Jiménez F, Barreto-Pérez D, Altamirano-Cardozo L, Loayza-Enríquez B, Farfán-García J (2021). Health status evaluation of type 2 diabetes patients from two hospitals of northern Peru. Rev Cuerpo Med HNAAA.

[24] Miranda Mesías R Creencias en salud de las personas con diabetes mellitus tipo 2 Hospital I Naylamp de ESSALUD Chiclayo 2018.

[25] Tan NC, Tan AM, Yeo JP, Rethink Study Group (2003). RETHINK An observational cross sectional study on the uptake of insulin therapy among type 2 diabetes patients with secondary drug failure treated in primary care. Asia Pac Fam Med.

[26] Jenkins N, Hallowell N, Farmer AJ, Holman RR, Lawton J (2010). Initiating insulin as part of the Treating To Target in Type 2 Diabetes (4-T) trial an interview study of patients' and health professionals' experiences. Diabetes Care.

[27] Polonsky WH, Fisher L, Guzman S, Villa-Caballero L, Edelman SV (2005). Psychological Insulin Resistance in Patients With Type 2 Diabetes The scope of the problem. Diabetes Care.

[28] Nakar S, Yitzhaki G, Rosenberg R, Vinker S (2007). Transition to insulin in Type 2 diabetes family physicians' misconception of patients' fears contributes to existing barriers. J Diabetes Complications.

[29] Nadasen DM, Naidoo M (2012). Patients with type 2 diabetes and difficulties associated with initiation of insulin therapy in a public health clinic in Durban. S Afr Fam Pract.

[30] Phillips A (2007). Starting patients on insulin therapy diabetes nurse specialist views. Nurs Stand.

[31] LaManna J, Litchman ML, Dickinson JK, Todd A, Julius MM, Whitehouse CR (2019). Diabetes Education Impact on Hypoglycemia Outcomes A Systematic Review of Evidence and Gaps in the Literature. Diabetes Educ.

[32] Ramírez LPA, Barrón AG, Valero KS (2012). Concepciones Culturales Sobre Insulinoterapia De Pacientes Diabéticos Tipo 2. Rev Salud Publica y Nutr.

[33] Liu Q, Tie L (2019). Preventive and Therapeutic Effect of Ganoderma (Lingzhi) on Diabetes. Adv Exp Med Biol.

[34] Hatami E, Ghalishourani SS, Najafgholizadeh A, Pourmasoumi M, Hadi A, Clark CCT (2021). The effect of spirulina on type 2 diabetes a systematic review and meta-analysis. J Diabetes Metab Disord.

[35] Dos Santos KC, Bueno BG, Pereira LF, Francisqueti FV, Braz MG, Bincoleto LF (2017). Yacon (Smallanthus sonchifolius) Leaf Extract Attenuates Hyperglycemia and Skeletal Muscle Oxidative Stress and Inflammation in Diabetic Rats. Evid Based Complement Alternat Med.

[36] Semlitsch T, Engler J, Siebenhofer A, Jeitler K, Berghold A, Horvath K (2020). (Ultra-)long-acting insulin analogues versus NPH insulin (human isophane insulin) for adults with type 2 diabetes mellitus. Cochrane Database Syst Rev.

[37] Ding H, Fatehi F, Russell AW, Karunanithi M, Menon A, Bird D (2018). User Experience of an Innovative Mobile Health Program to Assist in Insulin Dose Adjustment Outcomes of a Proof-Of-Concept Trial. Telemed J E Health.

[38] Miranda A, Garcia DO, Sánchez C, Warren C (2021). Health and Type 2 Diabetes Perspectives of At-Risk, Mexican-Origin Males (HD-MxOM) a Qualitative Study. J Racial Ethn Health Disparities.

[39] Organización mundial de la Salud (2020). Nota de prensa 1 de junio 2020. La COVID 19 afecta significativamente a los servicios de salud relacionados con las enfermedades no trasmisibles.

