# Supplementary material for: Myths and beliefs about insulin therapy in patients with diabetes mellitus and their family caregivers from a hospital in northern Peru, 2020
Source: Rev Peru Med Exp Salud Publica. 2023 Mar 30;40(1):42–50. doi: 10.17843/rpmesp.2023.401.12210 (PMC10953673; doi:10.17843/rpmesp.2023.401.12210)
Supplement: Translation

# Translation: Mitos y creencias sobre la insulinoterapia en pacientes con diabetes mellitus y sus familiares de un hospital del norte del Perú, 2020

**Authors:** Jhoana Lorena Vera Ubillús, Blanca Katiuska Loayza Enríquez, Rosa Elizabeth Guarníz Lozano, Franco Ernesto León Jiménez

**Affiliations:** 1 Centro de Salud de Quirichima, Lambayeque, Perú.; 2 Universidad San Martín de Porres, Filial Chiclayo, Chiclayo, Perú.; 3 Universidad Católica Santo Toribio de Mogrovejo, Lambayeque, Perú.; 4 Vicerrectorado de Investigación Universidad Privada Norbert Wiener, Lima, Perú.

**Keywords:** Insulina, Diabetes Mellitus, Investigación Cualitativa, Creencias, Entrevistas Grupales

## Abstract

**Objetivo.:**

Analizar y explorar los mitos y creencias sobre la insulinoterapia en pacientes con diabetes *mellitus* y sus familiares cuidadores de un hospital general del norte peruano en el 2020.

**Materiales y métodos.:**

Se realizo un estudio con enfoque cualitativo, paradigma interpretativo y tipo de análisis temático. Se obtuvieron datos sociodemográficos y clínicos de las historias clínicas y se entrevistaron pacientes con diabetes, con uso de algún tipo de insulina por lo menos tres meses antes del estudio, y a sus familiares cuidadores. Los pacientes participaron de un grupo focal y de entrevistas a profundidad; los familiares participaron solo en entrevistas a profundidad.

**Resultados.:**

Participaron 12 pacientes con diabetes (11 con diabetes *mellitus* tipo 2); seis en el grupo focal y seis en las entrevistas a profundidad y siete familiares. Luego del análisis se obtuvieron cuatro categorías: 1) creencias relacionadas al inicio de tratamiento con insulina: *tratamiento de elección después del fracaso con otros fármacos, cura la diabetes, regula el azúcar, temor a los inyectables*; 2) creencias relacionadas al mantenimiento del tratamiento: *descompensación por no usar insulina, la insulina es necesaria para vivir*; 3) creencias relacionadas a terapias alternativas y costo: *uso de terapias alternativas, costo elevado de la insulina*; y 4) mitos relacionados al uso de insulina: *genera dependencia, dependencia para la administración de insulina, efectos negativos de la insulina.*

**Conclusiones.:**

Las creencias y mitos de los pacientes, en tratamiento con insulina, emergen desde el inicio del tratamiento y se mantienen con la evolución de este, siendo en muchas ocasiones reforzados por la cosmovisión de los familiares.

## INTRODUCCIÓN

La diabetes *mellitus* (DM) es un problema de salud pública. Según las estimaciones realizadas por la Federación Internacional de la Diabetes (IDF, por sus siglas en inglés), alrededor de 537 millones de personas vivían con este problema en el mundo en 2021, con una proyección de 643 millones para el 2030 y con costos directos cercanos al billón de dólares. La prevalencia en el Perú es de 5,9% (intervalo de confianza al 95%: 5,5‒8,2), donde aproximadamente el 90% de pacientes poseen DM tipo 2 (DM2) ^1^.

En los pacientes con DM2, el tratamiento con insulina es un componente importante dentro de la terapéutica. Aproximadamente, se estima que del 13 al 26% de estos pacientes usan algún tipo de insulina como parte de su esquema de tratamiento ^2^^,^^3^. La Asociación Americana de Diabetes (ADA, por sus siglas en inglés) recomienda el uso de insulina para pacientes con enfermedad no controlada (pérdida de peso, hemoglobina glicosilada mayor de 10% o glicemias aleatorias de más de 300mg/dL) ^4^. 

En los pacientes que requieren insulina, la adherencia al tratamiento es fundamental, sin embargo, no es óptima. Davies *et al.*, en una revisión sistemática realizada en 2013, halló una frecuencia de adherencia que osciló entre el 43 y 86% ^5^. En el Perú, Flores realizó un estudio descriptivo en Lambayeque en 100 personas con diabetes en 2020, utilizando la prueba de Morinski Green, donde halló una frecuencia de no adherencia del 29% ^6^. Esta situación, lleva consigo un control glucémico deficiente y complicaciones clínicas con incremento de la mortalidad ^7^. 

Existe evidencia que respalda que los programas educativos estructurados pueden tener un impacto en personas con DM que usan insulina, mejorando el control metabólico, sin producir hipoglicemia. Hermmans *et al.*, en un ensayo clínico abierto realizado en 2017, evaluaron la efectividad de un programa educativo orientado a la autogestión en comparación con la educación estándar y halló un mejor control glicémico sin incremento de hipoglicemia ^8^. Asimismo, Yorke *et al.* realizaron una revisión sistemática de 36 estudios que incluyeron a 11,880 pacientes en 2017, donde reportan que las intervenciones educativas y estructuradas reducen la morbimortalidad y los eventos de hipoglicemia ^9^. Por otro lado, Iquize *et al.*, en otra revisión sistemática realizada en 2017, encontraron que la educación estructurada mejoraba la calidad de vida, pero no los valores de hemoglobina glicosilada ^10^. Esta discrepancia podría deberse, entre otros motivos, a diferentes perspectivas y opiniones sobre el uso de insulina en los pacientes ^11^. 

Para asegurar el éxito de las intervenciones educativas, debe tenerse en cuenta la perspectiva del paciente y de los familiares cuidadores, en especial en los pacientes que poseen múltiples comorbilidades y daño de órgano blanco. Conocer que piensan, sienten y que mitos y/o creencias tienen en relación con su enfermedad y sobre todo a la medicación que reciben, es crucial. Los programas educativos, en su mayoría, no consideran ese aspecto. En estudios cuali-cuantitativos, se ha demostrado que, identificando los mitos y creencias en una primera instancia, se pueden desarrollar estrategias educativas que llegarían a mejorar los niveles de hemoglobina glicosilada ^12^^-^^14^. Estos aspectos son más importantes con el tratamiento con insulina en términos de su aplicación subcutánea, el cálculo de la dosis y sus eventos adversos, los cuales, pueden hacer que el paciente y la familia no acepten el tratamiento ^15^. Se debe considerar que los miedos, expectativas, mitos y creencias pueden variar de un contexto a otro. Por otro lado, el soporte del entorno familiar en una enfermedad crónica y discapacitante como la DM2, es fundamental. Sin embargo, los cuidadores enfrentan una realidad compleja: dificultades para el entendimiento de la patología, creencias, nivel educativo, sentimientos de incertidumbre y falencias del sistema de salud para dar soporte en estilos de vida saludables ^16^^,^^17^. 

Ante la ausencia de datos sobre la perspectiva del paciente y del familiar cuidador, es importante generar información útil para gestionar mejoras en el programa de enfermedades crónicas no transmisibles en hospitales públicos del Perú. Por ello, se planteó analizar y explorar los mitos y creencias sobre la insulinoterapia en pacientes con DM2 y sus familiares cuidadores, de un hospital del norte peruano durante el año 2020.

**MENSAJES CLAVE**

Motivación para realizar el estudio. La adherencia al tratamiento con insulina en los pacientes diabéticos es subóptima y, además de determinar los factores clínicos y sociodemográficos, es preciso explorar la perspectiva del paciente y su entorno sobre el tratamiento.

Principales hallazgos. Los participantes tuvieron las siguientes ideas sobre el tratamiento con insulina: es necesaria para vivir si fracasan los medicamentos orales, cura la diabetes, regula el azúcar. Además, se evidenció temor por aplicarse un inyectable, a volverse dependientes y a la hipoglicemia. El costo de la insulina limita su uso y las plantas medicinales son percibidas como una alternativa.

Implicancias. La perspectiva del paciente y del familiar son fundamentales para la educación diabetológica.

## MATERIALES Y MÉTODOS

Para el reporte de los métodos y los resultados se utilizó el formato *Standards for Reporting Qualitative Research: A Synthesis of Recommendations* de la Academia de Medicina del año 2014 ^18^.

### Diseño

Se realizó un estudio con enfoque cualitativo, paradigma interpretativo, tipo análisis temático, desarrollado en tres momentos: descripción, reducción e interpretación. La moderadora de los grupos focales y responsable de las entrevistas a profundidad fue una licenciada en enfermería, investigadora cualitativa, docente universitaria, sin relación alguna con los pacientes ni con los familiares cuidadores (BLG). La responsable de hacer las anotaciones y grabado de las entrevistas fue una interna de medicina humana (LVU), sin relación con los pacientes y capacitada por la moderadora.

Para efectos del estudio se consideraron las siguientes definiciones de mito y creencia: i) mito definido según Taype, es una construcción social de distintos individuos que se propaga por la sociedad, carece de autor y trata de explicar un fenómeno, aunque no tenga un sustento científico, forma parte de una cultura, que tiende a considerarlos como verdaderos. Además, es una oposición a la verdad y la realidad que trata de explicar algo al margen de la racionalidad ^19^, y ii) creencia definida según Diez, es una idea reflexiva asumida por la sociedad y que el sujeto considera cierta y adopta como interpretación de su realidad; además, posee razones objetivas para considerarla así ^20^.

### Escenario y población de estudio

El estudio se llevó a cabo en el servicio de endocrinología del Hospital Belén de Lambayeque, del Ministerio de Salud (MINSA) del Perú, el cual es un establecimiento de salud de mediana complejidad, de la provincia y departamento de Lambayeque al norte del Perú, que atiende a pacientes con diabetes. El estudio se realizó entre enero y marzo del año 2020. 

Para el propósito del estudio, se incluyó a pacientes con DM tipo 1 y DM2, los que fueron seleccionados por conveniencia mediante saturación temática. Se incluyeron a pacientes con uso de cualquier tipo de insulina (rápida, intermedia, prolongada) por lo menos tres meses antes del estudio. Por otro lado, se excluyeron a aquellos pacientes con deterioro cognitivo demostrado, agudamente enfermos y con otras enfermedades crónicas importantes (tuberculosis, virus de la inmunodeficiencia humana). En los familiares, se incluyeron a quienes vivieran con ellos y que eran responsables de llevarlos a sus controles.

### Técnicas e instrumentos de recolección de información

Se recolectaron datos sociodemográficos, hemoglobina glicosilada y glicemia en ayunas de las historias clínicas (Material Suplementario). Se elaboraron dos guías de preguntas abiertas, revisados por un médico internista y una investigadora cualitativa. La primera guía de entrevista/grupo focal de seis preguntas abiertas, sirvió como orientación en el grupo focal de pacientes y en las entrevistas a profundidad al otro grupo de pacientes (Material Suplementario). La segunda guía de entrevistas fue de tres preguntas abiertas y sirvió como orientación en las entrevistas a profundidad a los familiares (Material Suplementario).

### Procedimientos

La selección de los participantes se llevó a cabo luego de su atención en el consultorio externo de endocrinología. Primero, se realizó el grupo focal, donde los pacientes con hipoacusia, ceguera o dificultad para movilizarse pudieran comunicarse. Esta entrevista se desarrolló en un consultorio cerrado y con apoyo de sus familiares, quienes los ayudaban a emitir sus respuestas. La duración de la sesión fue de 60 min y fue grabada mediante la grabadora de voz de un teléfono inteligente. Además, se realizaron notas de campo para luego incluir ambas fuentes en un texto (LVU). Por otro lado, las entrevistas a profundidad con los pacientes duraron 60 min cada una. La recolección de la información y la transcripción de las entrevistas a profundidad, siguieron el mismo procedimiento y las mismas características descritas para el grupo focal.

### Análisis de datos

A las entrevistas de los pacientes y familiares se les asignó un código: paciente: P mayúscula seguida de un número arábigo; familiar: F seguida de un número arábigo. Inicialmente, se realizó el proceso de degrabación de audios por uno de los autores (BLG), con experticia en este proceso.

El proceso del tratamiento de datos fue manual, siguiendo las fases del método de análisis temático propuesto por Braun y Clarke, el cual, es un método para identificar, analizar y reportar patrones (temas) dentro de datos recopilados empíricamente. Organiza mínimamente y describe detalladamente el conjunto de datos, además de interpretar aspectos del tema de investigación. En otras palabras, implica buscar a través de un conjunto de datos para encontrar patrones repetidos de significado ^21^. Las fases fueron: transcripción, reducción del texto y fase de descubrimiento, obteniéndose «temas» de estudio; posteriormente, se realizó la categorización: clasificación de datos según su contenido en común y vinculación hacia uno de los temas; finalmente, la fase de codificación: los fragmentos relevantes se asociaron a la categoría de un tema mediante un código (número). Mientras se realizaba el análisis, se redefinió la codificación y las categorías ajustándolas a los datos. Se triangularon los datos del discurso de los pacientes y el de los cuidadores. Esta labor fue realizada por uno de los autores (BLG).

### Aspectos éticos

El proyecto fue aprobado por la Dirección de Docencia del Hospital Provincial Docente Belén de Lambayeque y el Comité de Ética en Investigación de la Facultad de Medicina de la Universidad Católica Santo Toribio de Mogrovejo (Resolución N° 573-2019-USAT-FMED de fecha 31 de octubre del 2019), realizándose una enmienda a la misma mediante Resolución Nº 017-2022-USAT-FMED. Se utilizó un formato de consentimiento informado para los pacientes y otro para los familiares cuidadores, en los que se incluyó el objetivo del estudio, la confidencialidad de la información y la gratuidad de su participación. Finalmente, a los participantes, se les brindó una charla grupal sobre mitos y creencias en diabetes e insulinoterapia. Una copia del informe final fue entregado a la oficina de capacitación del hospital.

## RESULTADOS

Se incluyeron 12 pacientes con diabetes (11 con DM2), donde seis participaron de un grupo focal y seis pacientes de entrevistas a profundidad. Tambien, se entrevistaron a siete familiares cuidadores. Además de la información cuantitativa, se encontró que, de un total de 12 pacientes, seis tuvieron retinopatía, dos tuvieron nefropatía, uno tuvo una amputación previa y dos tuvieron neuropatía periférica. Ningún paciente estuvo en diálisis. Los datos cuantitativos se observan en la Tabla 1: edad, tiempo de enfermedad, años con insulina, glicemia en ayunas y hemoglobina glicosilada.

**Tabla 1:** Características de los participantes incluidos.

Características	Total (n=12)	%
Mujeres	10	84,0
Diabetes mellitus tipo 2	11	91,6
Nivel educativo		
Primaria	3	25,0
Secundaria	9	75,0
Complicaciones macrovasculares	3	25,0
Complicaciones microvasculares	7	58,3
Tipo de régimen		
Basal	6	50,0
Basal plus	3	25,0
Basal bolo	3	25,0
Tipo de insulina		
NPH sola	6	50,0
NPH y cristalina	6	50,0
Responsable de colocar la insulina		
Paciente	6	50,0
Familiar	6	50,0
Edad ^a^	51,5	42,5-58,0
Años con diabetes ^a^	7	5-13
Glicemia en ayunas (mg/dL) ^a^	170	150-186
HbA1c (%) ^a^	9	8,2-10,0
Meses con insulinoterapia ^a^	24	5-72

NPH: insulina de acción intermedia, cristalina: insulina de acción rápida, HbA1c: hemoglobina glicosilada.

a mediana y rango intercuartílico.

Asimismo, se sistematizaron cuatro categorías según el curso en el tiempo de la insulinoterapia: 1) creencias relacionadas al inicio de tratamiento con insulina; 2) creencias relacionadas al mantenimiento del tratamiento; 3) creencias relacionadas a terapias alternativas y costo; y 4) mitos sobre el uso de insulina (Tabla 2).

**Tabla 2:** Categorías generadas en el análisis de textos.

Categoría 1. Creencias relacionadas al inicio de tratamiento
*Es el tratamiento de elección tras el fracaso con otros medicamentos*
*Cura la diabetes*
*Nivela el azúcar*
*Temor a los inyectables*
Categoría 2. Creencias relacionadas al mantenimiento del tratamiento
*Miedo a descompensación por no usar insulina*
*Necesidad de la insulina para vivir*
Categoría 3. Creencias relacionadas a terapias alternativas y costo
*Terapias alternativas brindan el mismo beneficio que la insulina*
*Insulina es cara*
Categoría 4. Mitos relacionados al uso de insulina
*Dependencia para la administración de insulina*
*Genera dependencia*
*Efectos negativos*

### 1. Creencias relacionadas al inicio de tratamiento con insulina

Los entrevistados consideraron a la insulina como un segundo escalón en el tratamiento de su enfermedad, usualmente, precedido de un fracaso con antidiabéticos orales. También se mencionó el costo elevado del análogo de insulina y la necesidad de usar la insulina:

*Inicié con la insulina porque las pastillas no me bajaban la glucosa. Empecé con insulina <nombre comercial> y debido al costo luego cambié a NPH. En pacientes con diabetes tipo 2 la insulina se usa cuando las pastillas ya no hacen efecto. Mi glucosa era demasiado elevada a pesar de tomar pastillas y porque hacía desarreglos con la comida y bebía alcohol* (P4).

La diabetes es una enfermedad crónica, para la cual, aún no existe tratamiento definitivo. Las alternativas de tratamiento retardan la progresión y disminuyen las complicaciones de órgano blanco. Sin embargo, un paciente del grupo focal mencionó que la insulina podía curar la enfermedad:

*Mi hija dice que la insulina es una medicina que reemplaza el páncreas y mata la enfermedad* (P6).

Esta creencia también fue mencionada por un familiar:

*La insulina es una vitamina para que se cure de la diabetes* (F7).

Los familiares, juegan un rol fundamental en el apoyo o rechazo hacia el fármaco y sus opiniones o juicios pueden influir en el inicio del tratamiento:

*Mi esposo me hizo caso en que la insulina es para su bien porque llevamos 45 años de casados, le dije que si le recetan insulina es para su bien y debe estar tranquilo* (F1).

Pacientes y familiares mencionaron que la insulina puede controlar los niveles de glicemia:

*Uso insulina hace 10 años. Es un medicamento que regula el azúcar, es necesaria para que el hígado la procese de la sangre y los alimentos* (P7).

*La insulina ayuda a bajar la glucosa demasiado alta* (F6).

Otro hallazgo fue el temor a los inyectables, que podría condicionar el inicio de tratamiento y la necesidad de un familiar cuidador para ayudarlos con la administración:

*Al inicio tuve miedo porque las agujas con las que te ponen suero son grandes. Pero las agujas de la insulina no duelen porque son chiquitas. Me gustaría que la insulina no sea inyectable sino en jarabe porque es más fácil, no tengo que guardarla en hielo ni hincándome* (P6).

También, se hallaron las dificultades percibidas para colocarse un inyectable al empezar el tratamiento:

*Al inicio no sabía cómo inyectarme, no querían ponérmela ni en la farmacia. Posteriormente tuve que aprender, mi sobrina me enseñó, porque no quería molestar a los demás* (P8).

Los familiares también mostraron miedos al inicio del tratamiento:

*Tenía miedo al inicio porque veo muchos pacientes cojitos, y si no se curan les cortan las piernas* (F3).

Las creencias acertadas o erróneas de los pacientes se mantienen durante el tratamiento, por lo que, se generó una categoría que engloba las diferentes creencias con las que conviven y con las que gestionan su tratamiento.

### 2. Creencias relacionadas al mantenimiento del tratamiento

El reto para las personas con diabetes es lograr que no descontinúen la insulinoterapia. Algunos pacientes tienen miedo a descompensarse si suspendían el tratamiento:

*Tengo miedo a que no me inyecten y me ponga mal* (P1).

Los pacientes confían en el tratamiento, lo aceptan y opinan que les ayuda a vivir mejor:

*El doctor me dijo que podría permanecer con la insulina el resto de mi vida y lo acepté tranquilo porque mientras pueda estar con vida no hay problemas* (P5).

### 3. Creencias relacionadas a terapias alternativas y costo

Se aprecia el discurso sobre terapias alternativas; algunos entrevistados reportaban beneficios y la creencia que puede sustituir a la insulina:

*Me suelo cuidar con pastillas y productos naturales como Espirulina y café de hongo Ganoderma. Estos productos son igual a la insulina porque me controlan y me bajan el azúcar. Yo no creo que sea mi caso porque es suficiente con mi producto natural. En algún momento dejaré la insulina y solo tomaré lo natural porque hacen la misma acción* (P3).

*Hay remedios más naturales que son iguales, unas pajitas que venden en el mercado. Así ya no tendría que tener que estar inyectándose a cada rato* (F6).

Sin embargo, otros mencionan haberlos probado y dejado al poco tiempo pues no percibían mejoría:

*He usado yacón para bajar la glucosa, pero aun así tomando eso y otros remedios naturales no me hacen efecto* (P10).

Algunos pacientes mencionan que se les indicaba insulina que no tenía el hospital por lo que debían comprarlas, siendo el costo muy elevado:

*Me gustaría dejar de usar insulina porque cuesta mucho* (P3).

Los familiares comparten el mismo argumento:

*La insulina es cara, la compro porque a veces no hay en el SIS* (Seguro Integral de Salud) (F7).

### 4. Mitos relacionados al uso de insulina

En esta sección se aprecian los discursos de pacientes y familiares que entran dentro de la característica de mitos:

*Prefiero las pastillas porque son más fáciles. A veces me da miedo ponerle insulina, que se salga la sangre o le ponga mal* (F4).

Los pacientes opinaron que podían desarrollar dependencia, ya que al dejar de usar insulina surgen molestias.

*La insulina puede generar dependencia, porque al dejar de usarla sientes que debes volver a ponértela para estar bien* (P9).

Dentro de los discursos, se mencionó el querer dejar el tratamiento con la evolución de la enfermedad, pues a más años con diabetes, menos efecto de la medicación:

*La insulina actúa hasta cierto momento hasta que el cuerpo se deteriora producto de la enfermedad* (P11).

Los pacientes confunden síntomas de la progresión de la enfermedad por daño de órgano blanco con eventos adversos de la medicación:

*Dicen que la insulina es dañina y malogra órganos como por ejemplo la vista* (P8).

La explicación por parte del médico tratante puede ayudar a mejorar la adherencia:

*Al principio si tenía miedo de que me dañen algo como el estómago, pero después el endocrinólogo nos dijo que no me haría daño porque la aguja va a llegar preciso a lado de la grasita* (P4).

La hipoglicemia es una complicación aguda frecuente relacionada con dosis inadecuadas de insulina, su asociación con antidiabéticos orales, deficiente ingesta calórica, progresión de falla renal o procesos infecciosos. Muchas veces, el temor a esta descompensación genera que el paciente se administre dosis menores a las habituales:

*He tenido a la hipoglicemia como problema con la insulina. Por lo cual la dejé de usar un tiempo, pero luego me tomaron análisis y la volví a usar. Creo que me baja el azúcar cuando hago ejercicio y no como bien* (P12).

## DISCUSIÓN

Los pacientes entrevistados, a pesar de ser personas que tienen diabetes con complicaciones, no conocen los aspectos fundamentales de su cuidado y control, como el empleo de la insulina. Por ello, se encontraron varios mitos y creencias en relación con su uso.

La concepción de que puede *curarse* la enfermedad es una creencia hallada en el 11,7% de personas con diabetes que reciben atención en el primer nivel, según lo reportado en un estudio de México realizado en 2018 ^22^, y es una cifra similar a la hallada por León-Jiménez en un estudio realizado en pacientes del norte peruano en 2020 ^23^. Sin embargo, esta creencia no se halló en un estudio mixto en personas con diabetes atendidos en la Seguridad Social de Lambayeque en 2017 ^24^. En nuestro estudio, si se halló esta creencia, a pesar de haberse explorado en escenarios similares. Estos hallazgos dependen de múltiples factores como el nivel educativo, el nivel hospitalario donde la persona recibe atención, el tiempo de enfermedad y otras características. 

Asimismo, se encontró que algunos pacientes pensaban descontinuar el tratamiento por creer que está relacionado con ceguera, falla renal e inclusive, amputación de extremidades y no como producto de la progresión de su enfermedad. Este hallazgo es similar a los resultados encontrados en otros estudios como una revisión sistemática realizada por Ng *et al.*^15^. Este hallazgo muestra las limitaciones de los programas educativos de los hospitales públicos en lograr que el paciente conozca su enfermedad. La educación ideal es estructurada, secuencial, programada y medible, en grupos o de forma individual ^12^. Es necesario que los pacientes entiendan que el tratamiento mejora su vida y retrasa las complicaciones, ya que pone de relieve la importancia de la educación diabetológica. En el Perú, este aspecto es aún incipiente y no existe una forma estandarizada y sistemática de aplicación.

El hecho de que los pacientes sepan que la insulina *regula los niveles de azúcar en sangre* es positivo y alentador. Este resultado es similar al hallado por un estudio realizado por Tan *et al.,* en pacientes que no respondieron a terapia oral de la región de Asia en 2003, donde reportaron que el 59,7% respondieron que la insulina controlaba su glicemia ^25^ y un estudio cualitativo realizado por Jenkins *et al.* en un población inglesa en 2010, donde hallaron que los pacientes con el transcurrir de su enfermedad, aceptaban con mayor facilidad el uso de insulina ^26^. Este concepto también se evidenció en nuestro estudio. La denominada *resistencia psicológica a la insulina* es un concepto observado en aquellos que han experimentado un *fracaso personal* para controlar la enfermedad y es su última esperanza ^27^. Brod *et al.*, en un estudio mixto realizado en cinco países (Alemania, Suecia, Países Bajos, Reino Unido y Estados Unidos) hallaron que los pacientes consideraban a la insulina como terapia de último estadio y relacionaban el tratamiento como un *fallo personal*^14^. Esta concepción no es adecuada, pues si bien es cierto, la insulina está indicada en pacientes descompensados, otros pacientes se benefician de su uso desde el inicio ^4^. 

Sobre este punto, una posibilidad es que la educación diabetológica, aún incipiente en el Perú, no oriente a los pacientes para un uso temprano de insulina. Existen estudios que muestran que no sólo los pacientes, sino también el personal de salud, entre ellos, los médicos, tienen barreras para el uso de insulina. Este hecho se evidenció en la revisión sistemática realizada por Ng *et al.*, en 2015, donde se evaluaron los siguientes temas: conocimientos y habilidades deficientes (nueve estudios), inercia del médico (cinco estudios) y barreras del idioma (cuatro estudios) ^15^. Sin embargo, no se exploró esta posibilidad en este estudio. 

El temor para descompensarse por no usar insulina, como creencia, también fue un hallazgo. Al respecto, no hemos encontrado estudios que lo describan. Si bien es cierto no es una barrera, es un facilitador que debe ser direccionado por el personal de salud. Los pacientes que consideran a esta terapia como una herramienta útil, se adhieren con mayor frecuencia al tratamiento ^25^. 

La concepción de llegar a *depender* de la insulina, también se ha visto en otros estudios. Nakar *et al.* en un estudio de casos y controles que incluyó a 103 pacientes, hallaron que el grupo que necesitaba insulina en comparación con el que ya lo recibía, tenían más miedo a la adicción a la misma (39% *vs*. 20,8%; p<0,01) ^28^. Esta es una creencia que debe descubrirse y abordarse desde el inicio. 

La pérdida de autonomía y dependencia de los demás también fue un hallazgo, resultado similar al de otros estudios ^16^^,^^17^. Los pacientes deben ser educados desde el inicio, siendo que la presencia de un familiar que los asista no es una limitación sino una ventaja. La evaluación del componente emocional y mental de los pacientes y de quienes los cuidan es fundamental ^17^^,^^23^. La ceguera y la limitación funcional por secuelas neurovasculares son el escenario donde el apoyo de un familiar es imprescindible. 

La gran mayoría al comienzo manifestó rechazo a la insulina porque es inyectable. El *miedo a las inyecciones* se ha identificado como una barrera para el inicio del uso de insulina en varios estudios ^16^^,^^29^, siendo el dolor lo más mencionado. Existe evidencia de que esta preocupación tiende a desaparecer con el tiempo ^14^. 

El temor a la hipoglicemia fue referido por nuestros pacientes. Es un evento frecuente, para el cual, los pacientes y sus familiares cuidadores deben estar concientizados e informados. Este temor es válido y ha sido hallado en múltiples estudios ^29^^-^^31^. Al alta de la hospitalización, el personal de salud debe asegurarse que el paciente/familiar aprenda la dosis exacta, las formas de aplicación de insulina y los síntomas de alarma de hipoglicemia. LaManna *et al.* en una revisión sistemática de ocho estudios publicados entre 2014 y 2017 hallaron que la educación personalizada tenía impacto en el número de eventos de hipoglicemia y sus síntomas ^31^. Desconocemos si en este hospital o en otros, existe un protocolo estructurado para ello. 

Por otro lado, la incertidumbre de los pacientes los lleva a consumir terapias alternativas. Ramírez *et al.* realizaron un estudio descriptivo en México en 2021, donde reportaron que el 21% de los pacientes consumían alguna medicina tradicional, siendo la más frecuente, el *té de hierbas*^32^. La mención de *ganoderma* en nuestro estudio, es llamativa. *Ganoderma lucidum* es un producto que ha demostrado tener proteoglicanos y otros componentes con efecto hipoglicemiante ^33^; el mismo comentario para spirulina y diabetes; de hecho, existe una revisión sistemática de nueve estudios, que menciona su efecto beneficioso en lípidos y glicemia en ayunas ^34^. En relación con el yacón (*Smallanthus sonchifolius*), existe evidencia experimental en modelos animales acerca de su efecto antioxidante e hipoglicemiante ^35^. 

Además, se halló preocupación en relación con los costos de adquisición y aplicación de la insulina. El Seguro Integral de Salud incluye dentro de su petitorio nacional a la insulina NPH (Neutral Protamine Hagedorn) y a la insulina cristalina. Sin embargo, en los discursos se mencionó que no siempre se hallaban disponibles y debían comprarlas. Otros mencionaron el uso de insulinas no incluidas en el petitorio nacional o dispositivos de insulina precargada. Si bien es cierto, la hipoglicemia es levemente menos frecuente con estas insulinas, no hay diferencias en la eficacia para el control de la diabetes, en la aparición de complicaciones micro ni macro vasculares ni en la mortalidad ^36^. Adicionalmente, el ADA no los considera como la primera opción ^4^. 

Las preocupaciones por el monitoreo de la glicemia fue otro hallazgo. Una alternativa es el fortalecimiento del uso de la telemedicina como potente herramienta de monitoreo en atención primaria, la cual, ha demostrado ser eficaz ^37^. La posesión de un teléfono inteligente por muchos de los pacientes o cuidadores, es ahora una realidad. 

Entre las limitaciones metodológicas del estudio figuran el poco número de pacientes con DM tipo 1, y el mayor número de mujeres incluidas. En relación con estas dos limitaciones, si bien es cierto, el 90% de pacientes con DM son del tipo 2 ^1^, sería necesario desarrollar otro estudio en pacientes con DM tipo 1, que es más frecuente en jóvenes y niños. Este hecho permitiría explorar los mitos y creencias, pues el tratamiento de ellos es fundamentalmente con insulina, con inclusión de los padres quienes gestionan el uso de este medicamento en este grupo. Por otro lado, sería necesario también incluir más varones en futuros estudios pues su forma de pensar acerca de su enfermedad es distinta a las de las mujeres ^38^. Otras limitaciones son: el haber realizado un solo grupo focal en lugar de dos o más, para considerar un mejor espectro de participantes (sexo/tiempo de enfermedad), las preguntas aplicadas a pacientes en las entrevistas a profundidad fueron las mismas del grupo focal, la interpretación de los datos fue hecha por un solo investigador y el criterio de credibilidad (en el que se contrastan los resultados con los participantes) no se cumplió. Finalmente, debemos mencionar que esta información ha sido obtenida antes de la pandemia por la COVID-19. La cosmovisión del paciente/familiar podría haber cambiado con este nuevo contexto y sería pertinente explorarla. Además, la atención de varias estrategias de salud del MINSA se ha visto afectada por la pandemia, en especial de las enfermedades crónicas ^39^. 

En conclusión, los mitos y creencias de los pacientes con insulinoterapia emergen desde el inicio del tratamiento y se mantienen con la evolución de este, siendo en muchas ocasiones reforzados por la cosmovisión de los familiares y su presencia refuerza la importancia de la educación estructurada y proactiva, sabiendo que detrás de cada paciente existen concepciones sobre el proceso de salud y enfermedad, y que pueden ser distintos a los del personal de salud. No conocerlos puede impactar en la adherencia de los pacientes, sus familiares y en el pronóstico cardiovascular de los pacientes.
